# TiO_2_/PEG as smart anticorrosion and drug-eluting platforms in inflammatory conditions

**DOI:** 10.1016/j.heliyon.2024.e25605

**Published:** 2024-02-03

**Authors:** Sulieman Ibraheem Shelash Al-Hawary, Ruqayah Taher Habash, Munther Abosaooda, Ahmed Hjazi, Ebraheem Abdu Musad Saleh, Zahraa F. Hassan, Masoud Soroush Bathaei

**Affiliations:** a Department of Business Administration, Business School, Al al-Bayt University, Mafraq, Jordan; b College of Pharmacy, National University of Science and Technology, Dhi Qar, Iraq; c College of Pharmacy, The Islamic University, Najaf, Iraq; d Department of Medical Laboratory, College of Applied Medical Sciences, Prince Sattam bin Abdulaziz University, Al-Kharj 11942, Saudi Arabia; e Department of Chemistry, College of Science and Humanities in Al-Kharj, Prince Sattam Bin Abdulaziz University, Al-Kharj, 11942, Saudi Arabia; f College of Dentistry, Al-Ayen University, Thi-Qar, Iraq; g Department of Materials Engineering, Science and Research Branch, Islamic Azad University, Tehran, Iran

**Keywords:** Titanium implants, Composite coatings, Inflammatory, Smart drug delivery, Corrosion protection

## Abstract

The failure of a titanium implant is often attributed to inflammatory reactions following implantation. This study focuses on the synthesis of a polyethylene glycol (PEG) layer on porous titanium dioxide (TiO_2_) coatings using plasma electrolytic oxidation (PEO). This PEG layer serves as a foundation for a drug-eluting platform designed to respond to pH stimuli during inflammation. Betamethasone (BET), a widely used anti-inflammatory drug, was loaded onto the pH-responsive functional PEG layers. The application of the PEG-BET layer onto TiO_2_ coatings through the vacuum dip coating method resulted in a pH-sensitive sustained release of BET over a 30-day period. Notably, the release rates were 81% at pH 5.0 and 55% at pH 7.2. Electrochemical corrosion tests conducted in both normal and acidic inflammatory solutions demonstrated that duplex composite coatings offer superior protection compared to simple oxide coatings. In a pH 5.0 solution, corrosion current density measurements revealed values of 1.75 μA cm^−2^ (PEO/PEG-BET), 8.87 μA cm^−2^ (PEO), and 49.17 μA cm^−2^ (bare titanium). These results highlight the effectiveness of the PEO/PEG-BET layer in sealing pores within PEO coatings, subsequently reducing the infiltration of corrosive ions in inflammatory environments.

## Highlights

• Betamethasone (BET) loaded polyethylene glycol (PEG) layer is prepared on the plasma electrolytic oxidation (PEO)-modified Ti alloy.
• The PEO coating increased the corrosion resistance of Ti alloy in acidic inflammatory media.
• The PEG-BET layer helps to seal the pores and cracks of the PEO-treated Ti alloy.
• BET could be released sustainably from PEG under acidic inflammatory condition.
• Corrosion protection of PEO coating can be improved in inflammatory condition by PEG-BET layer.

## Graphical abstract

**Figure:**
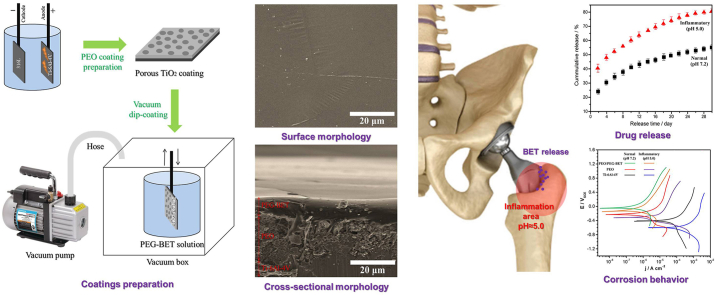

## 1 Introduction

The exceptional mechanical durability, favorable biocompatibility, and sufficient corrosion resistance exhibited by titanium (Ti) and its alloys make them an outstanding choice for metallic implants in biomedical applications. However, the aggressive environment within the human body can lead to corrosion of metallic implants [1]. Titanium's high affinity for oxygen allows it to form thin passivation layers exceeding nanometers in thickness, contributing to its remarkable corrosion resistance. Nevertheless, recent research has revealed the weak stability of this thin passive layer under inflammatory conditions [2].

Inflammation triggered after implantation can lead to the release of hypochlorous acid (HOCl) by osteoclasts and white blood cells, creating a local acidic pH region (pH ~ 5) around the implant. Consequently, surface treatment processes are essential to enhance the corrosion resistance of titanium implants in acidic inflammatory environments [3]. Long-term alleviation of inflammation may be achieved through the systemic delivery of anti-inflammatory drugs on the Ti surface.

Polymeric coatings have garnered attention for their ability to resist corrosive media and act as local drug delivery systems for anti-inflammatory agents [4,5]. The development of stimuli-responsive and smart drug-eluting implants aligns with the growing popularity of precision medicine and personalized pharmacotherapy [6]. In response to pH triggers caused by inflammation, the stimuli-responsive system releases its bioactive payload, achieving the intended local concentrations required for therapeutic effect and suppressing inflammatory reactions [7].

However, the adhesion of drug-loaded polymers to metallic substrates is generally weak, leading to explosive releases. Therefore, an intermediate coating is necessary for better adhesion of these layers to the Ti implant [8]. The Plasma Electrolytic Oxidation (PEO) technique, as an innovative electrochemical process, involves the anodic oxidation of Ti at higher voltages than the dielectric breakdown of the passive film, followed by microdischarges and the formation of ceramic TiO_2_ coatings comprising porous outer and compact inner layers [9]. Previous investigations demonstrated the efficacy of PEO coatings in enhancing the corrosion resistance of titanium-based biomaterials in challenging acidic environments encountered in biological settings [10].

The porous and rough structure of PEO coating is ideal for the mechanical interlocking of the polymer layer, creating a duplex coating [8,11]. Polyethylene glycol (PEG) is well-known for developing antifouling coatings on Ti implants [12]. The low molecular weight, high stability, anti-inflammatory, and osteogenic properties of betamethasone (BET) make it a promising candidate for bone regeneration [13,14]. While PEG has been explored only as an electrolyte additive in coatings for aluminum substrates in PEO coatings, its utilization as a protective top layer on PEO coatings in biomedical applications represents a novel and innovative approach, offering promising possibilities for enhanced corrosion performance by filling defects [15]. Moreover, PEG distinguishes itself with inherent pH-responsive properties [16], making it a dynamic and versatile component in the development of advanced drug-eluting systems. This study introduces a pioneering approach, presenting a pH-responsive drug-eluting system utilizing PEO/PEG coatings on titanium, aiming to achieve prolonged, stable BET release while ensuring robust corrosion resistance in inflammatory environments.

## 2 Materials and methods

### 2.1 Coatings preparation

The substrates were fashioned from a readily available cylindrical rod composed of Ti–6Al–4V (15 mm in diameter, adhering to Grade 23 specifications, sourced from Hermith GmbH). These substrates underwent meticulous refinement using SiC paper abrasives, progressing gradually to a 1200-grit finish. The Ti substrates were then subjected to oxidation using a PEO setup, which involved a DC power supply (Kikusui PWR-400H) maintaining a constant current density of 75 mA cm^−2^, with a 50% duty cycle, over a duration of 60 min. This oxidation process took place in a solution containing 10 g L^−1^ of potassium silicate (K_2_SiO_3_, sourced from Merck) and 3 g L^−1^ of potassium hydroxide (KOH, obtained from Sigma-Aldrich).

**Fig. 1:**
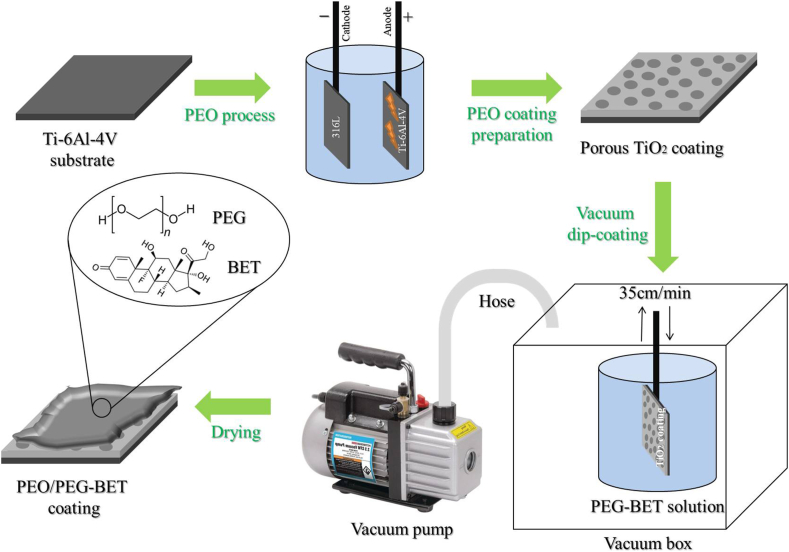
Schematic overview of the preparation of PEO and PEO/PEG-BET coatings on a Ti–6Al–4V substrate.

For the preparation of BET-loaded PEG, initially, 125 μL of triethylamine (Merck) were mixed with 0.98% PEG (Mw = 1000 g mol^−1^, Sigma-Aldrich) in 10 mL anhydrous toluene (99.8%, Merck) for 1 h. Subsequently, 2 mL BET (Zahravi) was slowly added to the solution and stirred for 10 min on the stirrer. The PEO-treated samples underwent a vacuum dip-coating procedure in a prepared solution at room temperature. The sample was withdrawn at a speed of 35 cm min^−1^ and then heat-treated at 40 °C for 24 h to partially densify the coating while ensuring no polymer degradation occurred. A visual representation of the coating preparation process is depicted in Fig. 1.

### 2.2 Coatings characterization

The surface and cross-sectional morphologies of the coatings were analyzed using field-emission scanning electron microscopy (FESEM, HITACHI S-4800). To determine the mean coating thickness and surface roughness, six measurements were conducted with a portable thickness and roughness gauge (PHYNIX-FN). The coatings' porosity was evaluated using ImageJ software, involving the analysis of FESEM micrographs to determine the percentage of porosity within the coatings.

X-ray diffractometer (XRD, Siemens-D500) with Cu Kα (λ = 1.54056 Å) radiation source under an angle of 20–80° was employed to examine the phase compositions of the coatings. Attenuated total reflectance Fourier transform infrared (ATR-FTIR, Nicolet iS5) was utilized to characterize the chemical composition of surfaces in the range between 4000 and 400 cm^−1^. For this analysis, samples of the PEO/PEG-BET-coated substrates were meticulously collected, thoroughly cleaned to eliminate surface contaminants, and securely mounted on the ATR crystal for direct contact. Baseline measurements were taken, followed by ATR-FTIR analysis.

The adhesion strength of duplex coatings was tested using a portable adhesion tester (PosiTest-AT-A50). Drug release studies were conducted on duplex-coated samples in triplicate by immersing them in separate 8 mL solutions: normal phosphate-buffered saline (PBS, Tamad Azma) with a pH of 7.2 as a simulated normal medium, and PBS+150 mMol HCl with a pH of 5.0 as a simulated inflammatory medium. The samples were shaken at 37 °C (120 rpm). To maintain system variables, 4 mL of the leaching solution were removed at specific times, and a similar volume of fresh solutions was added. The amount of released BET was detected using an ultraviolet–visible spectrophotometer (Shimadzu UV-3101PC).

For corrosion studies, a potentiostat (PAR, 263A) was employed to conduct electrochemical impedance spectroscopy (EIS) and potentiodynamic polarization (PDP) studies in normal and inflammatory media at 37 °C. The studies used a conventional cell with Ag/AgCl, platinum, and working electrodes. Anodic and cathodic polarization curves were generated by transitioning from the open-circuit potential (OCP) to the anodic and cathodic sides, employing a scan rate of 0.333 mV s^−1^. Electrochemical impedance spectroscopy (EIS) assessments were conducted over a frequency range spanning from 105 to 10-2 Hz, employing a sinusoidal voltage with an amplitude of 5 mV at the OCP.

## 3 Results and discussion

**Fig. 2:**
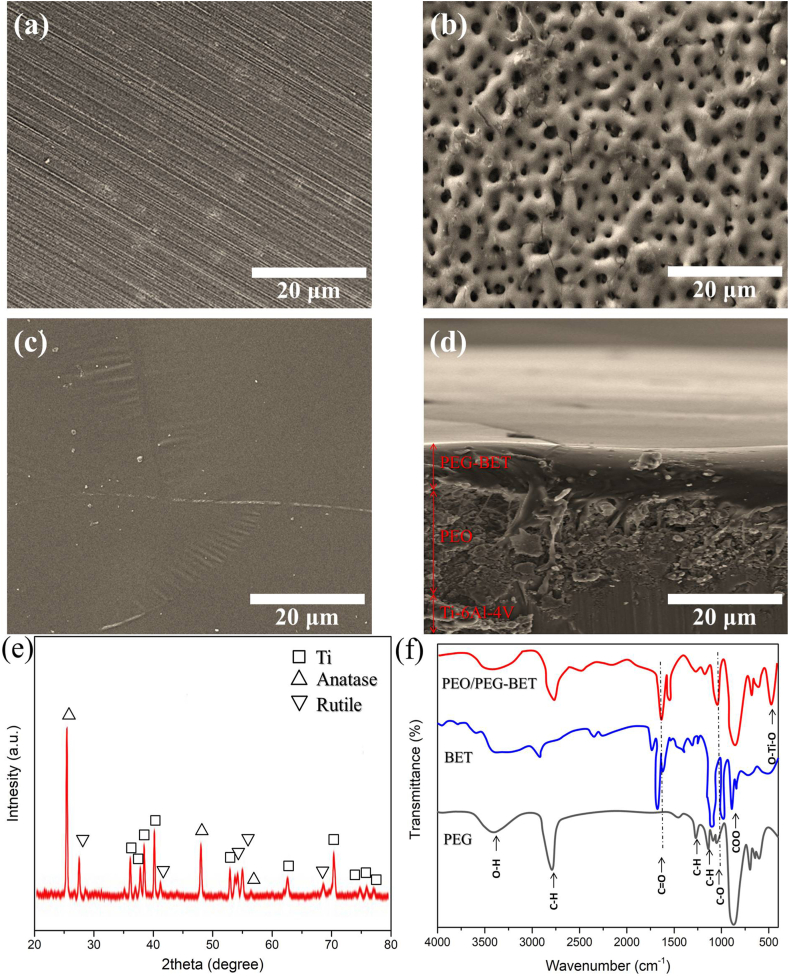
FESEM surface characteristics of (a) polished Ti–6Al–4V substrate, (b) PEO-Coated, (c) PEO/PEG-BET-coated samples, and (d) cross-sectional morphology of the PEO/PEG-BET coating. additionally, (e) presents the XRD pattern of the PEO coating, while (f) displays the ATR-FTIR spectra of PEG, BET, and the PEO/PEG-BET coating.

Fig. 2a and b presents the surface morphology of untreated and PEO-treated Ti samples observed through FESEM. A distinctive feature of these coatings is the presence of a porous, pancake-like layer observed on the PEO-coated surface. The top surface morphology of the PEO coating reveals a random distribution of micropores, ranging in size from 0.5 to 1 μm. This microstructure arises from the electron avalanche phenomenon triggered by electrical micro-discharges within the oxide layer. When a substantial electron current passes through the discharge channel, adjacent oxide materials undergo melting due to the pressure exerted by the emitted gases [17].

The surface and cross-sectional morphologies of PEO/PEG-BET are depicted in Fig. 2c and d, respectively. The cross-sectional micrograph of the PEO coating is also provided in Fig. S1 for reference, offering insights into its internal structure and thickness. In Fig. 2d–a polymer layer smoothly covers the crater-like surface treated with PEO, demonstrating uniform treatment based on surface morphology. Elongated ribbons of PEG-BET traverse the thickness of PEO coatings, illustrating the material's elastic nature.

The PEO/PEG-BET coating exhibited measurements of 31 ± 4 μm in thickness and 0.9 ± 0.3 μm in surface roughness. In comparison, the PEO coating had thickness measurements of 19 ± 3 μm, with a surface roughness of approximately 3.2 ± 0.7 μm. Porosity assessments using ImageJ software revealed a porosity of 38.42% for PEO and 0.22% for PEO/PEG-BET. Duplex coatings demonstrated reduced surface roughness, attributed to the PEG-BET layers covering microcracks and micropores in PEO coatings. The adhesive strength of the PEO/PEG-BET coating was measured at 41 MPa, meeting the ASTM1147-F standard, which requires a surface coating on biomedical material to have adhesion strength of at least 22 MPa [18].

The inclusion of the XRD test aimed to validate the crystalline structure of the oxide layer formed during the PEO coating process. According to the XRD pattern (Fig. 2e) for the PEO coating, characteristic peaks corresponding to JCPDS No. 96-900-9087 and JCPDS No. 96-900-4143 indicate the presence of rutile- and anatase-TiO_2_ phases, the primary components of the PEO coating. These phases are generated during the electrochemical oxidation in the PEO process at the interface between the Ti substrate and the electrolyte.

Fig. 2f illustrates the FTIR spectrum of the PEG-BET layer on the PEO-coated Ti, with additional spectra for PEG and BET for comparison. Distinctive peaks for PEG at 1090, 1453, and 2870 cm^−1^ indicate C–O stretching, C–H bending, and C–H stretching, respectively [12]. Characteristic BET peaks include 806 cm^−1^ for O–H bend, 1665 cm^−1^ for COO stretching vibration, and 1727 cm^−1^ for C=O stretching vibration [13]. Additionally, a peak at 510 cm^−1^ corresponds to the presence of TiO2 compounds generated by the PEO process beneath the PEG-BET layer [19].

**Fig. 3:**
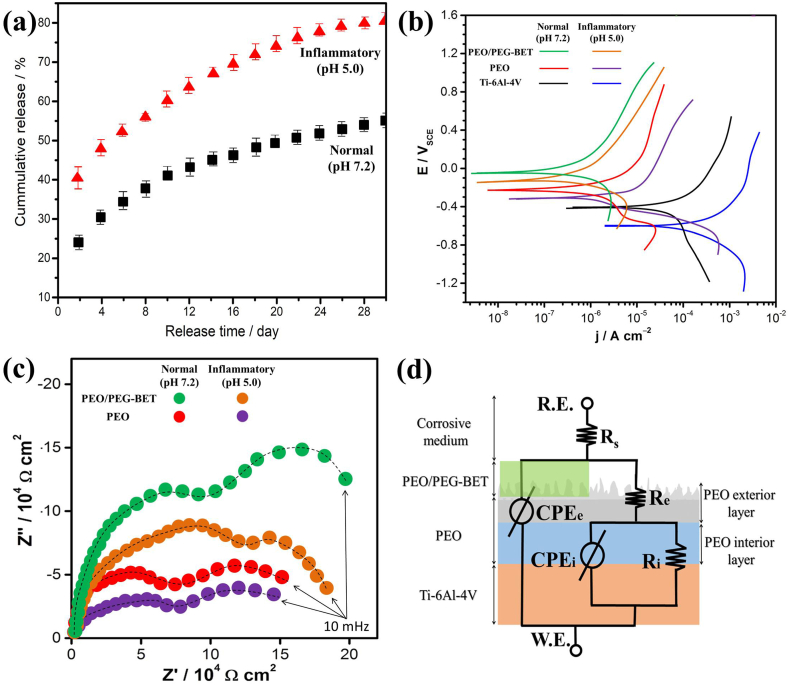
(a) PEG-BET release on PEO-coated Ti with time in normal and inflammatory solutions, (b) PDP curves, and (c) Nyquist plots of PEO, and PEO/PEG-BET coatings in normal and inflammatory solutions at 36.5 °C. (d) Proposed EC used for fitting of EIS data.

Fig. 3a shows the percentage cumulative release profiles for BET from the PEG layer on PEO-coated Ti at pH values 7.2 and 5.0. Under both conditions, the PEG-BET layers applied to the PEO coatings displayed favorable drug release patterns, characterized by an initial rapid release after a prolonged release extending for nearly 30 days. It is worth noting that when pH was reduced from 7.2 to 5.0, the BET-loaded PEG demonstrated a remarkable increase in cumulative BET release (>70% over 30 days). Upon exposure to an acidic inflammatory medium, BET drug molecules are rapidly and efficiently released from the PEG, whereas in the normal medium, drug release occurs slowly and steadily. In a similar manner, Yang et al. has recently reported that the release rate of paclitaxel/curcumin-loaded pH multistage responsive micelles was significantly accelerated after acid-induced detachment of the PEG layer [20].

Corrosion behavior of bare, PEO and PEO/PEG-BET coatings was also evaluated by PDP and EIS in Fig. 3b–and c, respectively. To ascertain the corrosion current density (i_corr_), corrosion process potential (E_corr_), and slopes of the cathodic and anodic curves (-β_c_ and β_a_), Tafel extrapolation was employed on the PDP curves within both the Tafel anodic and cathodic regions. We employed the Sterne-Geary equation to ascertain the resistance of polarization (R_p_) values [21]. To calculate the corrosion rate, Faraday's law was applied, converting the corrosion current density into a corrosion rate. This fundamental electrochemical principle allowed us to quantify the rate of corrosion for our samples [22]. Electrochemical parameters of PDP curves are listed in Table 1. Generally, the high corrosion resistance of the samples can be characterized by the high R_p_ and low i_corr_ values [21], ranked as follows: bare < PEO coating < PEO/PEG-BET coating. Moreover, it can also found that the corrosion resistance of all samples decreased in acidic inflammatory medium. According to the diameter of the capacitive loops in Nyquist plots (Fig. 3c), the PEO/PEG-BET-coated Ti had a larger loop radius than PEO, indicating high protection efficiency in both media [2,3]. The Bode modulus plots are also shown in Fig. S2. In all conditions, the Bode modulus of the PEO/PEG coating consistently exhibits higher values compared to the PEO coating. The obtained equivalent circuits (EC) from EIS fitting are shown in Fig. 3d, and the corresponding fitting data is summarized in Table 2. In EC, R_e_ signifies the resistance of the exterior porous layer, which is connected in parallel to the constant phase element (CPE)_e_ of the coating, whereas R_i_ denotes the resistance of the interior barrier layer, also connected in parallel to the constant phase element (CPE)_i_. The lower R_e_ value compared to R_i_ in both media indicates that the inner barrier layer determines the corrosion resistance of the entire coating system [23,24]. Furthermore, it is evident that the outer porous layers of composite PEO/PEG-BET-coated surfaces exhibited higher resistance compared to those of PEO-coated Ti. The reason for this is that corrosive media can easily penetrate the porous PEO coating due to its porous nature. Upon deposit of the PEG-BET layer over the PEO coating, a decrease in pores occurs, thereby increasing the number of pathways for corrosive agents' penetration [25].

**Table 1:** The extracted parameters from PDP curves of Ti–6Al–4V substrate, PEO, and PEO/PEG-BET samples in normal and inflammatory solutions.

Sample	PDP test's solution	E_corr_ (mV)	β_a_ (mV.dec^−1^)	β_c_ (mV.dec^−1^)	i_corr_ (μA.cm^−2^)	R_p_ ( × 10^4^ Ω cm^2^)	C_R_ (mm.year^−1^)
Ti–6Al–4V	Normal (pH 7.2)	−417	623	−274	8.26	0.82	0.1412
PEO		−206	576	−343	2.14	5.57	0.0363
PEO/PEG-BET		−54	471	−484	0.081	24.76	0.0013
Ti–6Al–4V	Inflammatory (pH 5.0)	−572	682	−471	49.17	0.32	0.8358
PEO		−329	721	−511	8.87	3.04	0.1508
PEO/PEG-BET		−154	602	−683	1.75	7.19	0.0297

**Table 2:** Parameters for fitting^a^ the EIS within the electrical circuits.

Sample	EIS test's solution	R_s_ (Ω.cm^2^)	(CPE-T)_e_ (S^n^. Ω^−1^.cm^−2^)	n_e_	R_e_ (kΩ.cm^2^)	(CPE-T)_i_ (S^n^. Ω^−1^.cm^−2^)	n_i_	R_i_ (kΩ.cm^2^)
PEO	Normal (pH 7.2)	21.11	1.77 × 10^−5^	0.85	7.48	1.54 × 10^−5^	0.83	17.02
PEO/PEG-BET		23.68	5.98 × 10^−6^	0.95	18.97	3.72 × 10^−6^	0.81	31.56
PEO	Inflammatory (pH 5.0)	15.73	1.42 × 10^−5^	0.86	4.72	0.21 × 10^−5^	0.85	9.20
PEO/PEG-BET		14.24	2.38 × 10^−6^	0.93	10.35	8.66 × 10^−5^	0.91	20.08

a The chi-square value for EIS fitting was approximately χ^2^ = 1 × 10^−4^.

## 4 Conclusion

In summary, this study investigated the drug delivery and corrosion behavior of Ti biomaterials coated with a novel PEO/PEG-BET composite in both normal and inflammatory environments. The incorporation of PEG facilitated a pH-sensitive sustained release of BET over a 30-day period on PEO-coated Ti. Utilizing a PEG-BET coating on PEO-coated Ti demonstrated the potential for sustained release of an anti-inflammatory drug, particularly in conditions of acidic environments induced by implantation-related inflammation. In the context of acidic inflammatory environments, the PEG-BET layer showcased remarkable efficacy as a robust barrier, fortifying the defense against corrosive agents and intrusive ions that may permeate the minute pores of the outer PEO coating. This enhancement significantly bolstered the corrosion protection capabilities of titanium-based biomaterials, promising improved durability and performance for biomedical applications. The integration of PEG-BET presents a promising avenue for advancing the resilience and longevity of these critical materials, offering new possibilities for the next generation of biomedical implants.

## Data availability

The authors do not have permission to share data.

## CRediT authorship contribution statement

**Sulieman Ibraheem Shelash Al-Hawary:** Writing – review & editing, Writing – original draft, Supervision, Software, Methodology, Investigation, Data curation, Conceptualization. **Ruqayah Taher Habash:** Writing – original draft, Visualization, Resources, Funding acquisition, Conceptualization. **Munther Abosaooda:** Writing – original draft, Resources, Methodology, Funding acquisition, Formal analysis. **Ahmed Hjazi:** Software, Methodology, Formal analysis, Data curation. **Ebraheem Abdu Musad Saleh:** Writing – original draft, Software, Resources, Methodology, Funding acquisition. **Zahraa F. Hassan:** Writing – review & editing, Writing – original draft, Formal analysis. **Masoud Soroush Bathaei:** Writing – review & editing, Writing – original draft, Supervision, Resources, Methodology, Funding acquisition.

## Declaration of competing interest

The authors declare that they have no known competing financial interests or personal relationships that could have appeared to influence the work reported in this paper.
