# Supplementary material for: TiO2/PEG as smart anticorrosion and drug-eluting platforms in inflammatory conditions
Source: Heliyon. 2024 Feb 3;10(4):e25605. doi: 10.1016/j.heliyon.2024.e25605 (PMC10867653; doi:10.1016/j.heliyon.2024.e25605)
Supplement: Multimedia component 1 [file mmc1.docx]

**Supplementary data**

**TiO_2_/PEG as smart anticorrosion and drug-eluting platforms in inflammatory conditions**

Sulieman Ibraheem Shelash Al-Hawary^1^, Ruqayah Taher Habash^2^, Munther Abosaooda^3^, Ahmed Hjazi^4^, Ebraheem Abdu Musad Saleh^5^, Zahraa F. Hassan^6^, Masoud Soroush Bathaei^7,*^

^1^Department of Business and Biomaterials Risk Assessments, Business and Dentistry School, Al al-Bayt University, Mafraq, Jordan

^2^College of Pharmacy National University of Science and Technology, Dhi Qar, Iraq

^3^College of pharmacy, the Islamic University, Najaf, Iraq

^4^Department of Medical Laboratory Sciences, College of Applied Medical Sciences, Prince Sattam bin Abdulaziz University, Al-Kharj, Saudi Arabia

^5^Department of Chemistry, Prince Sattam Bin Abdulaziz University,College of Arts and Science, Wadi Al-Dawasir, Saudi Arabia

^6^College of Dentistry, Al-Ayen University, Thi-Qar, Iraq

^7^Department of Materials Engineering, Science and Research Branch, Islamic Azad University, Tehran, Iran

*Corresponding author's emails: [ms.bathaei@gmail.com](mailto:ms.bathaei@gmail.com)

**
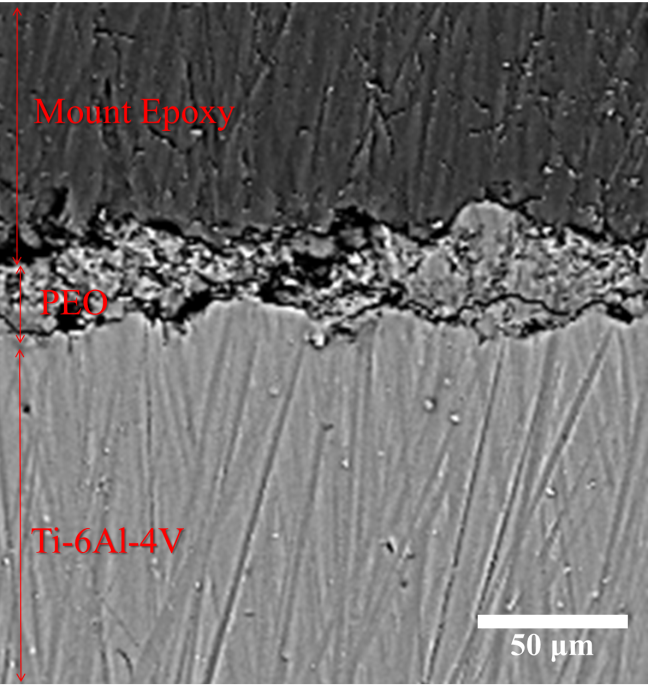
**

Figure S1. Cross-sectional micrograph of PEO coating on Ti-6Al-4V substrate

**
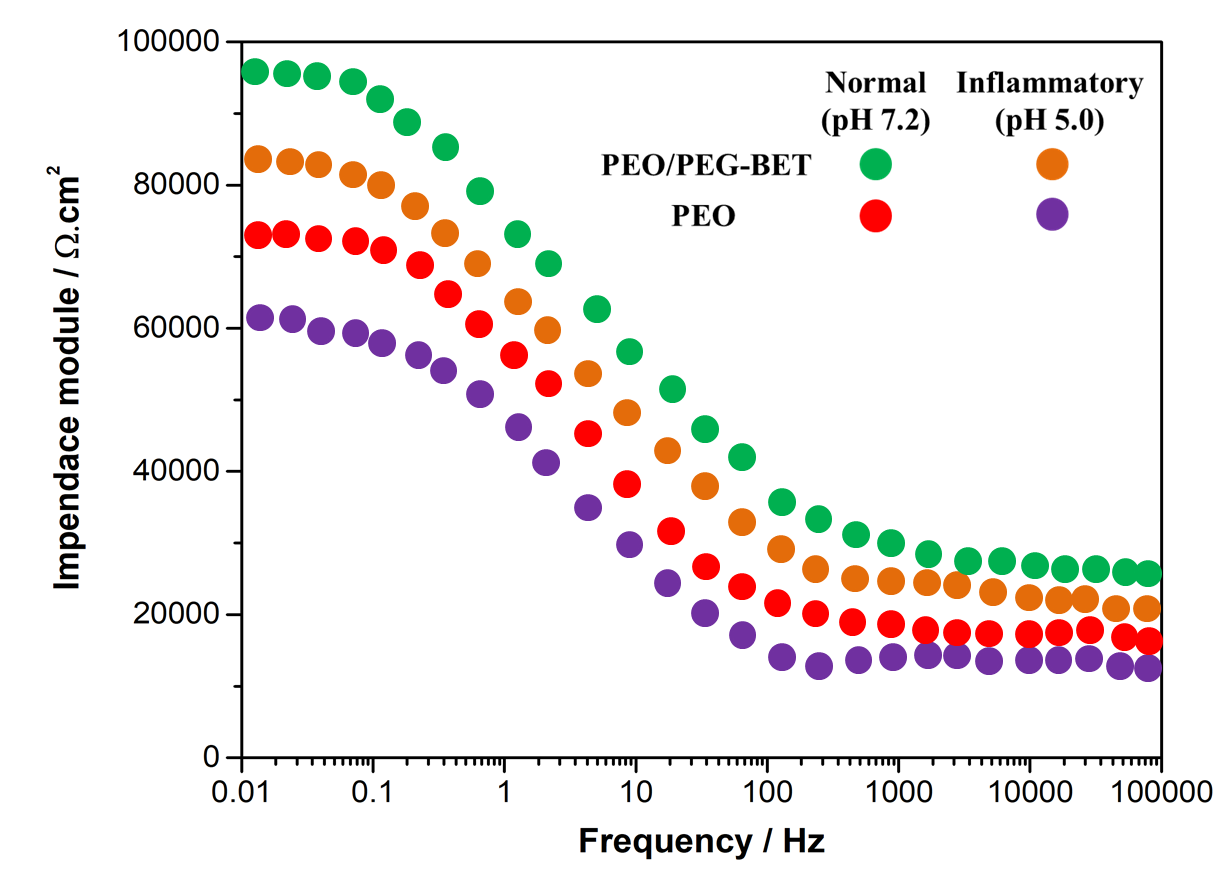
**

Figure S2. (a) Bode modulus plots of uncoated Ti-6Al-4V substrate, PEO, and PEO/PEG-BET coatings on a Ti-6Al-4V substrate in simulated normal and inflammatory solutions at 36.5 °C.
